# Frailty and physical performance in the context of extreme poverty: a population-based study of older adults in rural Burkina Faso

**DOI:** 10.12688/wellcomeopenres.15455.1

**Published:** 2019-09-11

**Authors:** Miles D. Witham, Justine I. Davies, Till Bärnighausen, Mamadou Bountogo, Jennifer Manne-Goehler, Collin F. Payne, Lucienne Ouermi, Ali Sie, Mark J. Siedner, Guy Harling

**Affiliations:** 1 AGE Research Group, NIHR Newcastle Biomedical Research Centre, Newcastle University, Newcastle, UK; 2 MRC/Wits Rural Public Health & Health Transitions Research Unit (Agincourt), University of the Witwatersrand, Johannesburg, South Africa; 3 Institute of Applied Health Research, University of Birmingham, Birmingham, UK; 4 Heidelberg Institute of Global Health, Medical Faculty and University Hospital, University of Heidelberg, Heidelberg, Germany; 5 Africa Health Research Institute, KwaZulu-Natal, South Africa; 6 Department of Global Health and Population, Harvard T.H. Chan School of Public Health, Boston, MA, USA; 7 Harvard Center for Population and Development Studies, Harvard University, Cambridge, MA, USA; 8 Centre de Recherche en Santé de Nouna, Nouna, Burkina Faso; 9 Division of Infectious Diseases, Massachusetts General Hospital, Boston, MA, USA; 10 School of Demography, Research School of Social Sciences, Australian National University, Canberra, Australia; 11 Institute for Global Health, University College London, London, UK

**Keywords:** Frailty, physical performance, prevalence, activities of daily living, sub-Saharan Africa

## Abstract

**Background: **Little is known about the prevalence of frailty and about normal values for physical performance among older individuals in low-income countries, in particular those in sub-Saharan Africa. We describe the prevalence of phenotypic frailty, and values and correlates of several physical performance measures in a cohort of middle-aged and older people living in rural Burkina Faso, one of the world’s poorest communities.

**Methods: **We analysed data collected from participants aged over 40 in Nouna district, Burkina Faso. We measured handgrip strength, four metre walk speed, chair rise time, and derived the Fried frailty score based on grip strength, gait speed, body mass index, self-reported exhaustion, and physical activity. Frailty and physical performance indicators were then correlated with health and sociodemographic variables including comorbid disease, marital status, age, sex, wealth and activity impairment.

**Results:** Our sample included 2973 individuals (1503 women), mean age 54 years. 1207 (43%) were categorised as non-frail, 1324 (44%) as prefrail, 212 (7%) as frail, and 167 (6%) were unable to complete all five frailty score components. Lower grip strength, longer chair stand time, lower walk speed and prevalence of frailty rose with age. Frailty was more common in women than men (8% vs 6%, p=0.01) except in those aged 80 and over. Frailty was strongly associated with impairment of activities of daily living and with lower wealth, being widowed, diabetes mellitus, hypertension, and self-reported diagnoses of tuberculosis or heart disease. With the exception of grip strength, which was higher in women than prior international normative values, women had greater deficits than men in physical performance.

**Conclusions: **Phenotypic frailty and impaired physical performance were associated as expected with female sex, co-morbidities, increasing age and impaired activities of daily living. These results support the use of frailty measurements for classification of ageing related syndromes in this setting.

## Introduction

Population ageing is now a reality in countries with low incomes and historically low life expectancy; particularly in sub-Saharan Africa ^1^, where improved control of infectious diseases has started to translate into rapid gains in life expectancy ^2^. Understanding the epidemiology of ageing in such countries is essential if limited resources are to be deployed to best support healthy ageing, and ensure that increased longevity provides individual and societal dividends ^3^.

Frailty is at the heart of understanding both healthy ageing and the impact of ill-health in old age. Frailty has been conceptualised as a state of reduced homeostatic reserve, leading to increased vulnerability to stressors ^4, 5^. In high- and middle-income countries, it is a powerful predictor of a diverse range of adverse outcomes for older people, including earlier death, increased need for healthcare, disability, dependency, injury and the need for informal and formal social care ^5– 8^. As such, it forms a key intermediate phenotype between disease and disability, and in high-income countries, it identifies a group of vulnerable older people who are most likely to benefit from Comprehensive Geriatric Assessment – an evidence-based, holistic approach to health and social care management for older people ^9^.

Although most work on frailty has been conducted in high-income countries, an emerging body of work supports the applicability of the concept in low and middle income countries (LMICs) ^10^. Recent studies from South Africa ^11^ using the Fried frailty criteria, and from Tanzania ^12, 13^ using both the Fried criteria and a cumulative deficit approach, have characterised frailty in older people. A review of studies in LMICs, including Brazil, Mexico, China and Russia, also demonstrated high rates of phenotypic frailty in community-dwelling older adults, ranging from 17 to 44%, depending on the definition and population ^14^. Given the disparity in prevalence between methods, settings and countries, data are required that are sufficiently distinct to capture country and culture-specific variations, using tools validated within the region of interest. Very little work has been done in the lowest income countries of sub-Saharan Africa, particularly in West Africa, where 14 out of 17 countries reside in the lowest development index category ^15^. A combination of poverty, weak healthcare infrastructure and competing demands in tackling communicable diseases, childhood and maternal health pose particularly difficult challenges to governments and healthcare providers seeking to support healthy ageing ^16, 17^.

There is consequently a need to ensure that widely used frailty tools – which were developed in high-income countries with very different populations, cultures and healthcare systems - are appropriate for use in local populations. Cumulative deficit frailty scores require collection of data on a large number of health domains – data that are not often readily available in many healthcare systems in LMICs. In contrast, phenotypic frailty lends itself to measurement in both clinical and research practice even in resource-poor environments, as it does not require costly infrastructure or comprehensive laboratory testing. Individual measures of physical performance, such as walk speed, grip strength and chair rise time are useful as part of frailty assessments and powerful individual predictors of adverse outcomes in a wide range of older populations; such measures can be collected and used even in the absence of formal diagnoses which may be difficult to access in resource-poor settings. Summarizing these measures across the age range and between sexes, provides crucial normative data upon which to base the use of such tools in clinical practice. Indeed, frailty and physical performance data will provide site-specific information on population ageing, while identifying groups at high risk of frailty-associated poor outcomes and health indicators, who would potentially benefit from health and social care interventions and services to maintain health and function in older age.

In this paper, we aimed to 1) characterise the prevalence of frailty, defined using the Fried Frailty Score, 2) describe the range and characteristics of commonly used measures of physical performance, and 3) assess the performance of frailty and physical performance scales and determine the correlates of frailty and physical performance in middle aged and older adults in the Nouna region of Burkina Faso, which ranks among the most rural regions of a country that falls in the lowest 2% of development globally.

## Methods

### Population description

The data used in this analysis were collected as part of the Centre de Recherche en Santé de Nouna (CRSN) Heidelberg Aging Study, hereafter abbreviated as CHAS. CHAS was conducted at the Nouna Health and Demographic Surveillance System (HDSS) site in Boucle du Mouhoun province, north-western Burkina Faso. The HDSS comprises the town of Nouna and 58 surrounding villages with a total population of ~107,000, and has been run by (CRSN) since 1993 ^18^. Most inhabitants are Muslim or Roman Catholic, although some are Protestant or Animist; several different ethnic groups are represented. The HDSS site is predominantly rural, comprising dry orchard savannah. Subsistence farming and cattle raising are major economic activities. Annual per-capita income is extremely low in the Nouna area, averaging only $400 per year, and school attendance averages only 1.2 years per person. These levels are lower than the average for Burkina Faso; figures in 2017 were $1650 and 1.5 years respectively.

### Survey

CHAS was a cross-sectional study of adults aged 40 years and older. The sample for CHAS was drawn from the 2015 HDSS household census using a stratified random sample of adults living in unique households within the HDSS. We sampled 4000 potential participants with the expectation of 25% non-response due to mortality since the prior census, inadequate mobility to participate or individuals declining to participate, with a target of 3000 responses. In the six villages with fewer than 50 adults aged over 40, all adults were selected. In the remaining communities, households containing at least one person aged over 40 were randomly selected, and then a potential respondent aged over 40 was randomly selected from each of these households.

Eligibility criteria for CHAS were: (i) being over 40 years of age; (ii) being primarily resident in a Nouna HDSS household for at least the past six months; and (iii) being willing and able to give informed consent to participate.

We collected data between May and July 2018 using tablet computers at respondents’ places of residence. Interviews were conducted either in French or translated into Dioula or Mooré, the most frequently spoken (but rarely written) local languages by fieldworkers; fieldworker training included translation practice. Blood was drawn by a trained phlebotomist also at respondents’ residences. Respondents with test results suggesting hypertension, diabetes mellitus, hyperlipidemia or anemia were recontacted and provided with a referral to the appropriate level of care with costs of care and travel provided.

### Consent

Ethical approval for CHAS was obtained from Ethics Commission I of the medical faculty Heidelberg (S-120/2018), the Burkina Faso Comité d’Ethique pour la Recherche en Santé (CERS) in Ouagadougou (2018-4-045) and the Institutional Ethics Committee (CIE) of the CRSN (2018-04). Oral assent was sought from village elders. Written informed consent was obtained from each participant; in cases of illiteracy, a literate witness assisted.

### Measures of physical performance and frailty

Three measures of physical performance were obtained. Walk speed was measured over a 4-metre course, marked out on level ground ^19^. Participants were asked to walk the course at their usual walking pace from a standing start. Two measures were obtained, the second attempt being performed in the reverse direction to the first. The time taken to complete the course was timed using a digital stopwatch and recorded to the nearest tenth of a second. The fastest time was used to derive walk speed in metres per second. Handgrip strength was measured using a Jamar Plus Digital Hand Dynamometer ^20^. Measurements were taken with the participant seated, the arm under test held at ninety degrees elbow flexion and with the shoulder and wrist in the neutral position. Two attempts were recorded from each hand, and the maximum value was used in this analysis. The time taken to rise and sit from a chair five times was recorded using a digital stopwatch. The test was performed using a standard chair without arms, and participants were asked to stand without using their arms to assist rising ^19^.

Thresholds for each domain of frailty were selected to be as close to those used in the original Fried derivation ^5^ as possible. Weight loss was defined as self-reported loss of >4kg over the last year (change in weight was reported as an integer). Low grip strength was defined as the lowest quintile of BMI-adjusted grip for each sex. Low walk speed was defined as the lowest quintile of height-adjusted walk speed over a 4-metre course for each sex. Low activity levels were defined as the highest quintile of self-reported hours of sitting per week for each sex. Self-reported exhaustion was measured using two questions from the eight-item CES-D scale ^21^: “Everything I did in the last week was an effort” or “I could not get going”. A positive response was defined as either question answered as applying for at least 3–4 days per week. Information on measurement of hours sitting per week, equations used to derive adjusted grip and walk speeds, and sex-specific cut-off values used to define the above variables are given in Table 1.

**Table 1.** Equations and cutoff values used to derive Fried frailty score.

	Men	Women
Mean grip strength (kg)	44.9	31.1
Equation for adjusted grip strength	= maximum grip - ((BMI - 21.6) * 1.17).	= maximum grip - ((BMI – 22.4) * 0.44).
Low BMI-adjusted grip strength	<37.1kg	<25.0kg
Mean walk speed (m/s)	1.045	0.888
Equation for adjusted walk speed	= walk speed - ((height in cm- 172) * 0.00579)	= walk speed - ((height in cm - 162) * 0.00849)
Low height adjusted walk speed	<0.821m/s	<0.720m/s
Self-reported weight loss	>4kg in last year	>4kg in last year
Self-reported exhaustion (2 questions)	Response of ‘3 to 4 days a week’ or more often to either question	Response of ‘3 to 4 days a week’ or more often to either question
Self-reported low activity levels (denoted by hours sitting per week)	>=34 hours	>=36 hours

BMI: Body mass index

The phenotypic frailty score was derived using a similar method to the original Fried score ^5^. Each domain (weight loss, low grip strength, low walk speed, self-reported exhaustion, low activity levels) scored one point, giving a final total of between 0 and 5 points. Individuals scoring zero were labelled as non-frail; 1–2 points labelled as prefrail, and 3 or more points labelled as frail. Individuals with missing data in one or more domains were labelled as ‘unable to calculate’; we describe how this group were handled in analyses below.

### Activities of daily living, comorbidities and sociodemographic variables

Basic activities of daily living (ADLs) - walking, transfers, toileting, dressing, bathing and eating - were captured via self-report. For all ADLs except walking, response options were: no difficulty, mild/moderate/severe difficulty, unable to do, or do not want to do. For walking, response options were: no difficulty, some difficulty, unable, or do not want to do. For this analysis, ADL impairment was classified as moderate or worse (some difficulty or worse for walking). Comorbidities were ascertained by a combination of self-report and objective measures. For a diagnosis of previous cancer, tuberculosis, chronic respiratory disease, stroke or heart disease, we relied on self-report of previous diagnosis or treatment. For HIV, we asked if participants had ever had a positive HIV test. We defined hypertension as either self-reported previous diagnosis or treatment, or a systolic blood pressure of >140mmHg, or a diastolic blood pressure of >90mmHg. Blood pressure was measured in the seated position after rest. Three measures were taken, and the mean of the second and third readings was used. We defined diabetes mellitus as either self-reported previous diagnosis or treatment, a random capillary glucose level of >200mg/dL, a fasting glucose level of >126mg/dL or an HbA1c level of >6.5%. Wealth was described by the construction of an asset index that included type and distance to water source, toilet characteristics, house room characteristics, cooking fuel, livestock, land area, electricity, furniture, watch, animal-pulled cart, cell phone, and bank account. Polychoric principal components analysis was used to derive an index as previously described ^22^ before division into quintiles. Marital status was obtained from self-report, divided into married/cohabiting, separated/divorced, widowed, or never married.

### Analyses

All analyses were conducted using SPSS v24 (IBM, New York, USA). A two-sided p value of <0.05 was taken to be significant for all analyses. Descriptive statistics were generated for baseline variables. Bivariate associations between frailty categories and other categorical variables were conducted using Pearson’s chi-squared test, or Fisher’s exact test where any cell value was five or less. To test whether disease and socioeconomic variables were associated with frailty status independent of age and sex, binary logistic regression models were run separately for marital status, wealth category and each diseases state, adjusting for age and sex. Between-group comparisons of continuous variables were conducted using Student’s t-test for normally distributed variables, and Mann-Whitney U test for non-normally distributed variables. Tests for trend across the categories non-frail, prefrail and frail for ADL impairments were performed using the Mantel-Haenszel test. Previous work has suggested that individuals with missing data have a prognosis similar to, or even worse than, those who are classed as frail ^11^. We therefore analysed those with missing data separately from those classed as frail in descriptive analyses, but included analyses using frail only, and frail combined with missing data, for univariate and multivariable analyses of association between baseline variables and frailty status.

To allow comparison of measures of physical performance in the CHAS cohort with other findings from the global literature, we additionally applied prespecified cutoffs for low walk speed (<0.8m/s), very low walk speed (<0.6m/s), prolonged chair stand time (>15s) and low handgrip strength (<27kg for men, <16kg for women) based on values used in previous studies and which are currently recommended in the European Working Group guidelines on sarcopenia diagnosis ^23, 24^.

## Results

**Figure 1.**
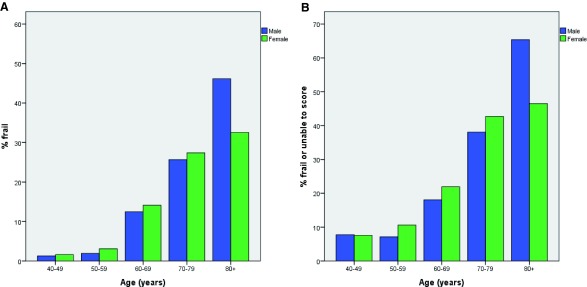
Frailty prevalence by age and sex. **A**. % with frailty (Fried score 3 or more). **B**. % with frailty (Fried score 3 or more) or unable to complete all components of frailty testing.

A total of 3998 individuals were approached to participate; 3028 (76%) agreed to participate and completed the baseline interview. 2973 individuals agreed to undergo measures of physical performance and were included in this analysis. Baseline details are given in Table 2. The prevalence of each frailty component, and overall prevalence of each frailty category, is given in Table 3. The overall prevalence of frailty in the study population was 7.1%, with a further 5.6% unable or unwilling to complete all of the components required to calculate a frailty score. The prevalence of frailty by age and sex is given in Figure 1A; Figure 1B shows similar data but aggregates those with frailty and those unable to have a frailty score calculated in the prevalence. In both cases, frailty was more prevalent at older age as expected; frailty was non-significantly more prevalent in women than in men except in the older old (those aged 80 and over) where frailty was significantly more common in men. Table 4 shows the distribution of individual frailty domains by age and sex, suggesting that the higher rates of frailty seen in the oldest men were due to greater prevalence of weak grip and low activity levels than that seen in the oldest women.

**Table 2.** Baseline characteristics (n=2973).

Mean age (years)(SD)		54.4 (11.0)
40–49 (%)		1236 (41.6)
50–59 (%)		856 (28.8)
60–69 (%)		546 (18.4)
70–79 (%)		267 (9.0)
80+ (%)		68 (2.3)
Female sex (%)		1503 (50.6)
Diseases	Diabetes mellitus	190 (6.4)
	Hypertension	1090 (36.7)
	Previous cancer diagnosis	14 (0.5)
	Previous tuberculosis	54 (1.8)
	Previously tested HIV positive	16 (0.5)
	Chronic respiratory disease	98 (3.3)
	Previous stroke	39 (1.3)
	Heart disease	165 (5.5)
Marital status (%)	Married/cohabiting	2246 (75.5)
	Separated/divorced	61 (2.1)
	Widowed	629 (21.1)
	Never married	34 (1.1)
	Missing data	3 (0.1)
Mean body mass index (kg/m ^2^) (SD)	Men	21.7 (3.5)
	Women	22.4 (4.8)
Mean walk speed (m/s) (SD)	Men	1.04 (0.27)
	Women	0.89 (0.23)
Mean grip strength (kg) (SD)	Men	44.9 (10.4)
	Women	31.1 (7.3)
Median chair stand time (IQR)	Men	13.0 (11.0 – 15.8)
	Women	15.0 (12.0 – 18.0)
Mean sedentary hours per week (SD)		25.6 (14.1)
At least one moderate ADL impairment (%)		851 (28.6)

Missing data: BMI: 4 missing. Chair stand: 160 missing. Walk speed: 43 missing. Grip strength: 35 missing ADL: Activities of Daily Living

**Table 3.** Prevalence of frailty score components and frailty categories.

Component		Frequency
>4 kg weight loss		256/2870 (8.9%) (103 missing)
Everything is an effort in past week OR Could not get going in past week		614/2972 (20.7%) (1 missing)
Lowest quintile walk speed (by sex, adjusted for height)		590/2930 (20.1%) (43 missing)
Lowest quintile grip strength (by sex, adjusted for BMI)		588/2935 (20.0%) (38 missing)
Highest quintile sitting time (by sex)		558/2962 (18.8%) (11 missing)
Frailty category (%)	Non-frail	1270 (42.7)
	Pre-frail	1324 (44.5)
	Frail	212 (7.1)
	Unable to score	167 (5.6)

BMI: Body mass index

**Table 4.** Prevalence of each frailty domain by age and sex.

	Low grip (%)		Low walk speed (%)		Weight loss (%)		Exhaustion (%)		Low activity (%)	
Age (years)	men	women	men	women	men	women	men	women	men	women
40–49	53/689 (8)	31/544 (7)	93/689 (13)	55/544 (10)	53/691 (8)	50/526 (10)	78/712 (11)	80/546 (15)	107/710 (15)	46/544 (8)
50–59	60/409 (15)	54/442 (12)	75/406 (18)	63/440 (14)	29/412 (7)	37/428 (9)	74/420 (18)	95/447 (21)	58/420 (14)	60/445 (13)
60–69	97/230 (42)	104/310 (34)	62/230 (27)	82/306 (27)	24/224 (11)	32/303 (11)	59/233 (25)	98/313 (31)	61/232 (26)	79/313 (25)
70–79	61/107 (57)	83/142 (58)	46/106 (43)	72/146 (49)	13/109 (12)	12/144 (8)	40/113 (35)	56/154 (36)	55/112 (49)	63/154 (41)
80+	21/24 (88)	24/38 (63)	15/24 (63)	27/39 (69)	3/25 (12)	4/42 (10)	14/26 (54)	24/43 (56)	17/24 (71)	17/43 (40)

Table 5 gives the prevalence of ADL impairments for each of the six basic ADLs by frailty category. ADL impairments were more common in those categorised as pre-frail than in non-frail, and more common again in those categorised as frail. Table 6 shows differences in grip strength, walk speed and chair stand time for those with and without ADL impairments. Significant differences were evident for most physical performance measures between those with and without each ADL impairment. Table 7 shows associations between a range of baseline characteristics and frailty. Those in the lowest wealth index category were more likely to be frail, as were those who were widowed. Frailty was associated with hypertension, diabetes, self-reported heart disease and previous tuberculosis after adjusting for age and sex, but interestingly not with self-reported previous cancer, stroke or a positive HIV test, although numbers reporting these diagnoses were low. In multivariable analysis, adjusting for age, sex, wealth and marital status, only hypertension and heart disease were independently associated with frailty, as shown in Table 8.

**Figure 2.**
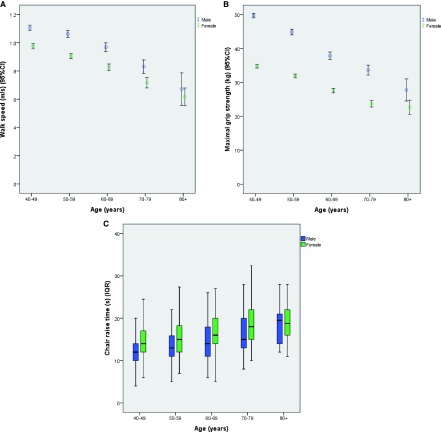
Physical performance by age and sex. **A**. Walk speed. **B**. Grip strength. **C**. Median chair stand time.

Physical performance measures were all significantly worse at higher ages as expected, and were worse in women than in men as shown in Figure 2. Using prespecified cutoffs to facilitate comparison with other studies of physical performance, we found that 63/1460 (4.3%) of men had a grip strength below 27kg and 21/1478 (1.4%) of women had a grip strength below 16kg. 358/1417 (25.2%) of men and 618/1396 (44.3%) of women had a chair stand time of >15 seconds. 135/1455 (9.3%) of men and 328/1475 (22.2%) of women had a walk speed of <0.8m/s; 44/1455 (3.0%) of men and 133/1475 (9.0%) of women had a walk speed below the more conservative threshold of <0.6m/s.

**Table 5.** Associations between frailty and significant ADL impairment (n=2972).

	Non-frail (n=1270)	Pre-frail (n=1324)	Frail (n=212)	P for trend *	Unable to score (n=167)	Whole cohort (n=2972)
Any ADL impairment (%)	264 (20.8)	390 (29.5)	130 (61.3)	<0.001	67 (50.3)	851 (28.6)
Difficulty walking (%)	216 (17.0)	337 (25.5)	119 (56.1)	<0.001	65 (38.9)	737 (24.8)
Difficulty dressing (%)	32 (2.5)	59 (4.5)	31 (14.6)	<0.001	23 (13.8)	145 (4.9)
Difficulty bathing (%)	50 (3.9)	95 (7.2)	38 (17.9)	<0.001	23 (13.8)	206 (6.9)
Difficulty eating (%)	61 (4.8)	67 (5.1)	25 (11.8)	0.004	14 (8.4)	167 (5.6)
Difficulty transferring (%)	44 (3.5)	82 (6.2)	43 (20.3)	<0.001	27 (16.2)	196 (6.6)
Difficulty toileting (%)	45 (3.5)	107 (8.1)	54 (25.5)	<0.001	30 (18.0)	236 (7.9)

*trend across non-frail, prefrail and frail ADL: Activities of Daily Living

**Table 6.** Associations between physical performance and level of ADL impairment (n=2972).

		Mean walk speed (m/s) (SD)		Mean grip strength (kg) (SD)		Median chair stand time (s) (IQR)	
		Men	Women	Men	Women	Men	Women
No significant ADL impairment		1.08 (0.26)	0.92 (0.22)	46.2 (9.8)	32.0 (7.0)	12.0 (11.0-15.0)	14.0 (12.0-18.0)
One or more significant ADL impairments		0.93 (0.28) *	0.82 (0.22) *	40.5 (11.3) *	29.2 (7.4) *	14.0 (11.0-17.5) *	16.0 (13.0-20.0) *
Difficulty walking	No	1.08 (0.26)	0.92 (0.22)	46.1 (9.8)	32.0 (7.0)	12.0 (11.0-15.0)	14.0 (12.0-18.0)
	Yes	0.90 (0.28) *	0.81 (0.22) *	40.0 (11.4) *	28.9 (7.5) *	14.0 (11.5-18.0) *	17.0 (13.0-20.0) *
Difficulty dressing	No	1.05 (0.26)	0.89 (0.22)	45.3 (10.3)	31.3 (7.2)	13.0 (11.0-15.1)	15.0 (12.0-18.0)
	Yes	0.91 (0.41) *	0.81 (0.25) *	37.1 (11.4) *	27.1 (7.5) *	13.0 (11.0-17.0)	17.0 (13.0-20.0) *
Difficulty bathing	No	1.05 (0.26)	0.89 (0.22)	45.4 (10.2)	31.3 (7.2)	13.0 (11.0-15.0)	15.0 (12.0-18.0)
	Yes	0.87 (0.33) *	0.82 (0.24) *	37.4 (10.8) *	28.5 (7.2) *	14.0 (12.0-20.0) *	17.0 (13.0-19.0) *
Difficulty eating	No	1.05 (0.27)	0.89 (0.23)	45.2 (10.3)	31.3 (7.2)	13.0 (11.0-15.1)	15.0 (12.0-18.0)
	Yes	0.91 (0.24) *	0.86 (0.20)	40.0 (11.6) *	28.2 (6.9) *	14.0 (12.0-16.5)	15.0 (12.5-19.0)
Difficulty transferring	No	1.06 (0.26)	0.90 (0.22)	45.4 (10.1)	31.3 (7.2)	13.0 (11.0-15.0)	15.0 (12.0-18.0)
	Yes	0.84 (0.34) *	0.79 (0.24) *	36.7 (11.6) *	27.7 (7.2) *	14.0 (12.0-19.5) *	16.0 (12.0-19.0)
Difficulty toileting	No	1.06 (0.26)	0.90 (0.22)	45.4 (10.2)	31.4 (7.1)	13.0 (11.0-15.0)	15.0 (12.0-18.0)
	Yes	0.82 (0.31) *	0.81 (0.25) *	37.6 (11.9) *	27.6 (7.5) *	14.0 (11.3-18.0) *	16.0 (13.0-19.0) *

*p<0.05 vs no impairment. ADL: Activities of Daily Living

**Table 7.** Associations between other baseline characteristics and frailty.

		Frail (n, %)	p	OR (95% CI)	p	Frail or unable to score (n, %)	p	OR (95% CI)	p
Marital status	Married or cohabiting	107/2246 (4.8)	<0.001	1 (-)	-	207/2246 (9.2)	<0.001	1 (-)	-
	Never married	1/34 (2.9)		0.59 (0.08 to 4.38)	0.61	3/34 (8.8)		0.95 (0.29 to 3.13)	0.93
	Separated/divorced	3/61 (4.9)		1.06 (0.33 to 3.43)	0.93	7/61 (11.5)		1.28 (0.58 to 2.86)	0.54
	Widowed	101/629 (16.1)		4.36 (3.12 to 6.11)	<0.001	160/629 (25.4)		3.47 (2.67 to 4.51)	<0.001
Wealth index (quintile)	1 (lowest)	67/589 (11.4)	<0.001	1 (-)	-	111/589 (18.8)	<0.001	1 (-)	-
	2	38/596 (6.4)		0.54 (0.36 to 0.82)	0.004	72/596 (12.1)		0.61 (0.44 to 0.84)	0.002
	3	32/592 (5.4)		0.46 (0.30 to 0.71)	0.001	50/592 (8.4)		0.41 (0.29 to 0.59)	<0.001
	4	33/608 (5.4)		0.46 (0.30 to 0.71)	<0.001	65/608 (10.7)		0.53 (0.38 to 0.74)	<0.001
	5 (highest)	42/587 (7.2)		0.62 (0.41 to 0.93)	0.02	80/587 (13.6)		0.70 (0.51 to 0.96)	0.03
Diabetes mellitus		20/192 (10.4)	0.07	1.54 (0.95 to 2.52)	0.08	35/192 (18.2)	0.04	1.45 (0.98 to 2.16)	0.06
No diabetes mellitus		187/2705 (6.9)				354/2705 (13.1)			
Hypertension		108/1090 (9.9)	<0.001	1.84 (1.39 to 2.44)	<0.001	191/1090 (17.5)	<0.001	1.88 (1.51 to 2.34)	<0.001
No hypertension		104/1883 (5.5)				188/1883 (10.0)			
Previous cancer		0/14 (0)	0.62 *	NC	NC	2/14 (14.3)	1.00 *	1.16 (0.26 to 5.23)	0.85
No previous cancer		206/2968 (6.9)				421/2968 (14.2)			
Previous TB		8/54 (14.8)	0.02	2.27 (1.06 to 4.88)	0.04	13/54 (24.1)	0.04	2.16 (1.14 to 4.08)	0.02
No previous TB		202/2956 (6.8)				419/2956 (14.2)			
Prev HIV positive test		0/17 (0)	0.63 *	NC	NC	1/17 (5.9)	0.50 *	NC	NC
No prev HIV positive test		197/2799 (7.0)				396/2799 (14.1)			
Chronic respiratory disease		8/101 (7.9)	0.70	1.14 (0.55 to 2.39)	0.72	15/101 (14.9)	0.86	0.94 (0.51 to 1.74)	0.84
No chronic respiratory disease		200/2886 (6.9)				411/2886 (14.2)			
Previous stroke		4/40 (10.0)	0.45	1.57 (0.55 to 4.47)	0.40	7/40 (17.5)	0.57	1.32 (0.55 to 3.19)	0.54
No previous stroke		208/2988 (7.0)				427/2988 (14.3)			
Heart disease		21/166 (12.7)	0.003	1.91 (1.18 to 3.10)	0.009	32/166 (19.3)	0.06	1.53 (1.01 to 2.30)	0.04
No heart disease		187/2837 (6.6)				398/2837 (14.0)			

Odds ratios adjusted for age and sex. *Fishers exact test. NC: Not calculable. TB: Tuberculosis

**Table 8.** Multivariable analysis - associations between baseline characteristics and frailty.

		Frail		Frail or unable to score	
		OR (95% CI)	p	OR (95% CI)	p
Age (per year)		1.00 (1.00 to 1.00)	0.97	1.00 (1.00 to 1.00)	0.97
Female sex		0.62 (0.43 to 0.90)	0.01	0.77 (0.58-1.02)	0.07
Marital status	Married or cohabiting	1 (-)	-	1 (-)	-
	Never married	1.81	0.57	1.03 (0.30 to 3.47)	0.97
	Separated/divorced	1.70	0.66	0.86 (0.19 to 3.94)	0.84
	Widowed	6.63	0.07	3.06 (0.89 to 10.52)	0.08
Wealth index (quintile)	1 (lowest)	1 (-)	-	1 (-)	-
	2	0.65 (0.41 to 1.02)	0.06	0.74 (0.52 to 1.05)	0.09
	3	0.51 (0.31 to 0.83)	0.007	0.46 (0.31 to 0.69)	<0.001
	4	0.57 (0.35 to 0.91)	0.02	0.59 (0.41 to 0.85)	0.005
	5 (highest)	0.60 (0.38 to 0.95)	0.03	0.69 (0.48 to 1.00)	0.05
Diabetes mellitus		1.14 (0.63 to 2.06)	0.66	1.22 (0.78 to 1.92)	0.39
Hypertension		1.70 (1.25 to 2.32)	0.001	1.71 (1.34 to 2.19)	<0.001
No previous cancer		NC	NC	1.18 (0.25 to 5.54)	0.83
No previous TB		1.23 (0.42 to 3.64)	0.71	1.34 (0.57 to 3.14)	0.51
Previous positive HIV test		NC	NC	NC	NC
Chronic respiratory disease		0.97 (0.40 to 2.33)	0.94	0.82 (0.40 to 1.70)	0.60
Previous stroke		2.02 (0.66 to 6.18)	0.22	1.43 (0.52 to 3.91)	0.49
Heart disease		1.94 (1.15 to 3.29)	0.01	1.64 (1.04 to 2.56)	0.03

NC: Not calculable. TB: Tuberculosis

## Discussion

In this sample of middle-aged and older people living in rural Burkina Faso, we found that frailty, as measured using the Fried phenotype was present in 7.7% of the population, was more common with advancing age, and was slightly more common in women than in men, with the exception of those aged 80 and over, where frailty became more common in men. Higher rates of ADL impairments were seen in those with frailty than with prefrailty or no frailty. Grip strength, chair stand time and walk speed were all worse with advancing age; grip strength (particularly for women) was very high compared to population data from other LMICs.

The prevalence of frailty that we found was similar to that found in rural South Africans of similar age (5.4 to 13.2%) in the Health and Ageing in Africa: A longitudinal study of an INDEPTH community (HAALSI) study ^11^. A recent study in those aged 60 and over in Tanzania found a prevalence of 14% ^12^, which was similar to the prevalence in those aged 60 and over in our sample. However, frailty prevalence at age 70–79 was high in our sample (30%); considerably higher than found in the same age group in Tanzania (16%) or in HAALSI (10–15%). Although frailty prevalence increased steeply with age as expected, our finding that frailty was more prevalent in men than in women in the oldest (80+) stands in contrast to most other populations studied. This finding appears to be driven by a higher prevalence of low activity levels and low grip strength in the oldest men, although the reasons for these findings require further study. Those aged 80 and over are rare in Burkina Faso, constituting 0.5% of the Nouna population in 2007 ^18^, and are likely to represent healthy survivors, yet despite this, frailty prevalence is very high in this group.

Grip strength was much higher in our cohort than that recently described in Tanzania ^12^, even when confining the comparison to those aged 60 and over. Similarly, grip strength for both sexes was much higher at each comparable age when compared to data from the HAALSI cohort in South Africa – approximately 10kg higher for those aged 40–49, and 5kg higher for those aged 80+ ^25^. Comparing grip strength with normative values from high income countries (HICs) and LMICs ^26^ showed that values in our cohort were similar to normative data from HICs, and considerably higher than that from many other LMICs in Africa and Asia. This finding was particularly striking for women, with mean grip at all ages well above the mean for UK normative data. This finding may be driven by the high proportion of women engaged in agrarian labour ^27^. Mean walk speed was faster for males and females in our cohort than that seen in HAALSI at each comparable age, or Tanzania (0.65m/s); it was also faster than most values seen in WHOSAGE in middle income countries (mean 0.61 to 0.88m/s) ^28^. Few data exist for chair stand times in LMICs, but values in our study for those aged 70–79 were comparable to those seen in a study of people aged 65 and over living in urban South Africa ^29^. The reasons for these high levels of physical performance require further study, but may be related to the high levels of lifetime physical activity in the agrarian lifestyle that most participants had lived for the majority of their lives. These differences may be accentuated by survivor bias; less healthy individuals, who tend to have lower physical performance, are less likely to survive given the lack of sophisticated healthcare infrastructure.

Despite these high levels of physical performance, frailty prevalence was no lower than in comparable LMICs; this is likely to reflect the methodology used to derive phenotypic frailty scores, which relies on identifying the lowest 20% of the study population for many of the components. This approach gives a higher prevalence of frailty than might be expected on the basis of the raw physical performance data. Two main methods have been proposed for identifying frailty: phenotypic scores and cumulative deficit scores. Phenotypic scores (e.g. the Fried frailty criteria ^5^) focus on physical frailty and are comprised of a small number of measures, such as physical function (walk speed and grip strength) weight loss, self-reported exhaustion, and self-reported low activity levels. In contrast, cumulative deficit models (e.g. Rockwood’s frailty index ^30^) tally a number of deficits across a wide range of organ systems. For instance, they compile diagnoses of medical diseases, deficits of biochemical testing and cognitive test scores, and low measures of physiological function such as lung function or muscle strength. Such cumulative deficit modelling approaches are thought to require at least 30 variables for a reliable score ^31^. We chose to use the Fried frailty approach for this study, partly to reduce respondent burden, but also because in the absence of healthcare personnel and infrastructure to support comprehensive diagnosis and recording, it is unlikely that sufficient deficits for the Frailty Index would be either diagnosed or collected in clinical practice in Burkina Faso. It is possible however that a frailty index approach would give a different prevalence of frailty, as it is less dependent on physical performance measures.

ADL impairment was more common in individuals with frailty compared to those who were pre-frail or non-frail. This finding is expected, is similar to that observed in other cohorts ^11^ and supports the validity of frailty measurement in the Burkina Faso cohort. Despite good walk speed and grip strength, ADL impairment was paradoxically very common, and this was despite a conservative approach to classifying ADL impairment. The reasons for this paradox are not immediately apparent. The dispersion of grip strength and walk speed was similar in our cohort to that seen in South Africa in HAALSI ^11^, but despite this, physical performance values worse than the cutoff values in the current European sarcopenia guidelines ^23^ were common. This suggests that although physical performance in the whole population was good, there was considerable heterogeneity, with at least some of the high prevalence of ADL impairment being attributable to poor physical performance. Some participants with ADL impairment clearly still had good physical performance, as shown by the mean walk speed, grip strength and chair stand times in those with ADL impairment. Further study is required to understand how the respondents in Burkina Faso understood the questionnaire, how they conceptualised activity limitation, and how they decided on their response to these questions.

Some other limitations require comment. We studied only one area of Burkina Faso, and thus our results may not generalise to other communities within the country. Currently, only cross-sectional data are available, but future waves of data collection should allow us to examine trajectories of physical performance and frailty, as well as allowing us to study how well frailty and physical performance predict future adverse outcomes. We had to rely on self-report for most disease states, as healthcare facilities to enable a full suite of investigations and diagnoses were not feasible to deliver within the confines of the CHAS study, and local healthcare facilities are limited in both scope and coverage.

Although Burkina Faso has one of the lowest levels of income of any country, life expectancy at birth has increased markedly in recent years – from 49.5 years in 1990 to 60.8 years in 2017 ^32^. In keeping with many other LMICs therefore, Burkina Faso is undergoing a rapid expansion in the number of older people in the population, and this trend is likely to continue as cohorts surviving childhood continue to age. Understanding the prevalence and correlates of frailty and physical performance is a key step in designing health and social care systems that can maintain physical function and ameliorate the effects of frailty on health, wellbeing and societal roles for older people. It is striking that rates of frailty even in this very poor community are significant; given the strong relationship between frailty and older age, number of people living with frailty are likely to increase as the population continues to age. Frailty is an important predictor of future disability and dependency. Preventing or treating frailty is an important goal to maintain health and function in older age – one that communities such as Nouna have only limited financial and technical resources to address. Interventions such as Comprehensive Geriatric Assessment (CGA) have been shown to improve health and function in high-income countries ^9^. The process of CGA provides a base from which interventions tailored to local needs and resources could be developed, and frailty provides an important basis for selecting who would be likely to benefit from such interventions. The association between cardiovascular disease (including hypertension) and frailty in this population is noteworthy and suggests one possible avenue for frailty prevention via control of cardiovascular disease. Population-based interventions including nutrition, physical activity, social support and healthcare systems all have a part to play in the maintenance of physical function; future work should investigate the relationship between these factors and the trajectory of physical function and frailty in very low-resource settings such as rural Burkina Faso.

## Data availability

Data are not publicly available as consent was not given by participants for data to be shared openly. This is in part because entire age cohorts of some villages are included in the dataset, potentially allowing for deductive disclosure with sufficient local information. For this reason, anonymized data is available from CHAS study data controllers only following signature of a data use agreement restricting onward transmission. Anyone wishing to replicate the analyses presented, or conduct further collaborative analyses using CHAS (which are welcomed and considered based on a letter of intent), should contact Dr Guy Harling ( g.harling@ucl.ac.uk) in the first instance.
