# Supplementary material for: Frailty and physical performance in the context of extreme poverty: a population-based study of older adults in rural Burkina Faso
Source: Wellcome Open Res. 2019 Sep 11;4:135. [Version 1] doi: 10.12688/wellcomeopenres.15455.1 (PMC7137808; doi:10.12688/wellcomeopenres.15455.1)
Supplement: Peer review history

# Peer review: Reviewer response for version 1

**DOI:** 10.21956/wellcomeopenres.16902.r36742

**Published:** 2020-04-06

**Authors:** Ruth E. Hubbard

**Affiliations:** 1 Centre for Health Services Research, Faculty of Medicine, The University of Queensland, Brisbane, Australia

The aim of this study was to describe the prevalence of frailty among people aged > 40 years  in rural Burkina Faso, sub-Saharan Africa.

 The paper is very well written. The introduction clearly articulates the rationale for undertaking the study. The cohort is moderately sized and participants comprehensively evaluated. A well validated measure of frailty (the Fried phenotype) was applied.

 While many of the study results are not surprising (e.g. the increase in frailty prevalence with advancing age; the association between frailty and co-morbidities/ADL impairment), frailty has not previously been explored in this population so establishing its prevalence and confirming its construct validation is a valuable contribution to the field.

 The discussion section comprises a detailed consideration of the results and careful contextualization with other relevant studies.

I confirm that I have read this submission and believe that I have an appropriate level of expertise to confirm that it is of an acceptable scientific standard.

# Peer review: Reviewer response for version 1

**DOI:** 10.21956/wellcomeopenres.16902.r37006

**Published:** 2019-11-25

**Authors:** Maria-Victoria Zunzunegui

**Affiliations:** 1 University of Montreal, Montreal, Canada

This is an excellent paper, original, well written and it contributes to the literature on aging in West Africa. I have some comments authors may want to take into account in the final version of the paper. 

1. Lifecourse, gender perspectives and selective survival could help explain the very high physical function of older men and women in Nouna. More contextual information could be given in the introduction or discussion: infant mortality and maternal mortality historical data would help to better understand aging in this extremely poor context. 
2. Selective survival should be seriously considered in international studies on aging in LMIC. The authors mention that life expectancy was 49.5 years in 1990. However, in 1990 most of the study cohort was older than 50 years of age, therefore they had survived the life expectancy they would have had if they had been born in 1990. Those born in 1938 are 80 years and over in 2018 when the study was conducted, and have more than doubled their life expectancy. In a small study conducted in women from North East Brazil, we found evidence for a strong survivor bias since telomeres of those women who had suffered the most economic and social adversity were longer than telomeres of women who had been exposed to less adversity ^1^. 
3. Reproductive history is related to physical performance in old age. Most of these women have had multiple childbirths from an early age and their chances to survive to old age were very small in a context of high maternal mortality and very limited post-natal care or care for chronic diseases. Surviving for women is at least equally hard as for men. Authors could cite previous research on the effects of reproductive history on physical performance and frailty. See for example: Pirkle *et al.* ^2^. And related papers by Dr Pirkle using data from the IMIAS Study.
4.  Lastly, women have more self-reported disability and cognitive deficits than men of comparable age in Burkina Faso. This gender difference in prevalence of self-reported disability may be larger than the difference in objectively measured physical performance of lower mobility and the gender difference may be gender-related since women have to perform the hardest physical work in every-day life. See for example: Onadja *et al*. ^3^.

I confirm that I have read this submission and believe that I have an appropriate level of expertise to confirm that it is of an acceptable scientific standard.

## References

[1] : Lifecourse Adversity and Telomere Length in Older Women from Northeast Brazil. *Rejuvenation Res*.2018;21(4) : 10.1089/rej.2017.1937 294-303 10.1089/rej.2017.1937 28482745

[2] : Early maternal age at first birth is associated with chronic diseases and poor physical performance in older age: cross-sectional analysis from the International Mobility in Aging Study. *BMC Public Health*.2014;14: 10.1186/1471-2458-14-293 293 10.1186/1471-2458-14-293 24684705PMC3977880

[3] : Gender differences in cognitive impairment and mobility disability in old age: a cross-sectional study in Ouagadougou, Burkina Faso. *Arch Gerontol Geriatr*.57(3) : 10.1016/j.archger.2013.06.007 311-8 10.1016/j.archger.2013.06.007 23827740
